# Ectoparasites of the Critically Endangered Giant Shovelnose Ray *Glaucostegus typus* in the Eastern Indian Ocean, with a Summary of the Known Metazoan Parasites

**DOI:** 10.1007/s11686-024-00918-8

**Published:** 2024-09-17

**Authors:** Jack Ingelbrecht, Karissa O. Lear, Alan J. Lymbery, Rebecca L. Bateman, Bradley M. Norman, Storm B. Martin, Travis Fazeldean, David L. Morgan

**Affiliations:** Centre for Sustainable Aquatic Ecosystems, Harry Butler Institute, Murdoch University, 90 South Street, Murdoch, WA 6150 Australia

**Keywords:** Copepoda, Giant guitarfish, Glaucostegidae, Gnathiidae, Hirudinida, Monopisthocotyla

## Abstract

**Purpose:**

This study examined the metazoan ectoparasites of the Critically Endangered giant shovelnose ray, *Glaucostegus typus*, in the eastern Indian Ocean.

**Methods:**

We screened 186 *G*. *typus* for ectoparasites in four coastal regions of Western Australia between 2020 and 2022: the Pilbara Region, Exmouth Gulf, Ningaloo Coast and Shark Bay.

**Results:**

Five parasite taxa were encountered on 186 *G*. *typus*: *Caligus furcisetifer* (Copepoda: Caligidae), *Dermopristis cairae* (Monopisthocotyla: Microbothriidae), *Branchellion plicobranchus* and *Stibarobdella macrothela* (Hirudinida: Piscicolidae), and praniza larvae of unidentified gnathiid isopod/s (Isopoda: Gnathiidae). Two of these species, *B. plicobranchus* and *S. macrothela*, are reported for the first time on *G. typus*. Only *C*. *furcisetifer* and *S*. *macrothela* were relatively common, encountered on 31% and 40% of *G*. *typus*, respectively. Gnathiids were observed infrequently, encountered on 13% of *G*. *typus*, and *D*. *cairae* and *B. plicobranchus* were scarce, encountered on 1% and 2% of *G*. *typus*, respectively. Intensity of infection for *C*. *furcisetifer* and gnathiids increased with host length. Likelihood of infection varied seasonally for *C. furcisetifer*, being considerably lower in summer, and regionally for gnathiids, being greatest at Shark Bay. Intensity and likelihood of infection for* S*. *macrothela* increased with host length and varied regionally, being greatest at Shark Bay.

**Conclusion:**

These findings improve our understanding of the downstream impacts for dependent parasites that might arise should populations of *G*. *typus* continue to decline.

**Supplementary Information:**

The online version contains supplementary material available at 10.1007/s11686-024-00918-8.

## Introduction

Parasites are important components of ecosystems, in terms of species richness, biomass and maintaining ecosystem function [1, 2]. However, parasites also likely comprise the majority of fauna that are threatened with extinction [3–6], being vulnerable to direct threats, such as anthropogenic impacts [6], and also indirectly, via coextinction with their hosts [5, 7, 8]. Parasites with density-dependent transmission and specificity for threatened hosts face the greatest risk of extinction, because transmission may decrease below the threshold required to maintain the parasite population as the host population declines [5, 9]. Predicting outcomes for dependent parasites following a decline in the host population necessitates an understanding of those taxa [10, 11], yet most parasites remain poorly understood and perhaps the majority are still unknown to science [12]. If they are to be considered in host conservation plans, it is imperative that parasites of threatened host species are identified [5].

The giant shovelnose ray, *Glaucostegus typus* (Anonymous [Bennett], 1830), is a large species of giant guitarfish (Glaucostegidae) that has suffered severe population declines and fragmentation throughout much of its range as a consequence of overfishing [13–16]. Collectively, the giant guitarfishes are one of the most imperilled vertebrate families, with all seven extant species classified as Critically Endangered on the International Union for Conservation of Nature’s (IUCN) Red List of Threatened Species [14, 17–22]. It is estimated that populations of *G*. *typus* have been reduced by more than 80% over three generations across the northern limit of its distribution in the Indo-West Pacific [14, 15]. Although it is unprotected throughout its entire range, *G*. *typus* is afforded some refuge in Australian waters, including along the mid to northern coastline of Western Australia, where it is common as far south as Shark Bay [14, 16, 23, 24].

The metazoan parasite fauna of *G*. *typus* is relatively well characterised and better investigated than any other glaucostegid, comprising 28 reported taxa (Table 1). Of these, 11 species are known only from *G*. *typus*, although three have been considered *species inquirenda* [25] and a further three are known only from Borneo, where identifications of *G*. *typus* are seemingly ambiguous [26]. These investigations are limited to studies of a single parasite species or group and there are no reports of parasites of *G*. *typus* from the expansive coastline of Western Australia in the eastern Indian Ocean. The aims of this study were to characterise the metazoan ectoparasites infecting *G*. *typus* in the eastern Indian Ocean, and to examine intrinsic (size and sex of the host) and extrinsic (season and region) factors influencing infections.

**Table 1:** Metazoan parasites previously reported from the giant shovelnose ray, *Glaucostegus typus* (Rhinopristiformes: Glaucostegidae)

Parasite	Microhabitat	Locality	Reference
Arthropoda: Copepoda: Siphonostomatoida: Caligidae			
*Caligus furcisetifer*	Body surface	Moreton Bay Q. Aus	Boxshall [35]
*Lepeophtheirus acutus*	Eye, buccal mucosa, claspers	Burgers’ Zoo, Netherlands	Kik *et al.* [64]
Arthropoda: Copepoda: Siphonostomatoida: Sphyriidae			
*Tripaphylus australis* as *Paeon australis*	Gills	Moreton Bay Q. Aus	Kabata [65]^b^
Myxozoa: Multivalvulida: Kudoidae			
*Kudoa hemiscylli*	Somatic muscle	Moreton Bay and Coral Sea: Heron Is. Q. Aus	Gleeson *et al.* [66]
Nematoda: Rhabditida: Ascaridoidea: Acanthocheilidae			
*Mawsonascaris australis*	Spiral valve, stomach	Q. Aus	Sprent [67]^b^
Platyhelminthes: Cestoda: Diphyllidea: Echinobothriidae			
Echinobothrium chisholmae*	Spiral valve	Coral Sea: Heron Is. Q. Aus	Jones and Beveridge [68]^c^; Olson *et al.* [69]
	Spiral valve	Gulf of Carpentaria: off Weipa NT Aus	Ivanov and Caria [70]
*Echinobothrium* cf *rhynchobati* = *Macrobothridium rhynchobati* = *Macrobothridium* sp.*	Spiral valve	Timor Sea: Lee Point/Shoal Bay NT Aus	Caira *et al.* [71]^c^; Olson and Caira [72]^c^; Olson *et al.* [69]
Echinobothrium tetabuanense*	Spiral valve	Sulu Sea: off Kampung Tetabuan, Sabah, Malaysian Borneo	Ivanov and Caria [70]^d^
Echinobothrium weipaense*	Spiral valve	Gulf of Carpentaria: off Weipa NT Aus	Ivanov and Caria [70]
Platyhelminthes: Cestoda: Lecanicephalidea: Lecanicephalidae			
*Floriparicapitus plicatilis*	Spiral valve	Moreton Bay Q. Aus	Cielocha *et al.* [73]
	Spiral valve	Gulf of Carpentaria: off Weipa NT Aus	Cielocha *et al.* [73]
	Spiral valve	Timor Sea: Fog Bay NT Aus	Cielocha *et al.* [73]
Platyhelminthes: Cestoda: Rhinebothriidea: Escherbothriidae			
*Stillabothrium amuletum* = Anthobothrium amuletum*,	Spiral valve	Moreton Bay Q. Aus	Butler [74]^e^
= Rhinebothriinae new genus 3 sp. n. 7 (see Reyda *et al.*, 2016)	Spiral valve	Timor Sea: Fog Bay, NT, Aus	Healy *et al.* [75]^c^
Platyhelminthes: Cestoda: Tetraphyllidea: Balanobothriidae			
Balanobothrium rhinobati*^a^	Spiral valve	Arabian Sea off Veraval, Gujarat, India	Jadhav and Shinde [76]^c^
Balanobothrium somnathai*^a^	Spiral valve	Arabian Sea off Veraval, Gujarat, India	Jadhav and Shinde [76]^c^
Balanobothrium veravalensis*^a^	Spiral valve	Arabian Sea off Veraval, Gujarat, India	Jadhav and Shinde [77]^c^
Platyhelminthes: Cestoda: Trypanorhyncha: Eutetrarhynchidae			
*Dollfusiella spinosa*	Spiral valve	Off Kampung Tetabuan, Sabah, Borneo	Schaeffner and Beveridge [78]^d^
*Dollfusiella spinulifera*	Spiral valve	Moreton Bay Q. Aus	Beveridge and Schaeffner [79]
= *Prochristianella spinulifera*	Spiral valve	Coral Sea: Heron Is. Q. Aus	Beveridge and Jones [80]^c^; Miquel and Świderski [81]^c^; Olson *et al.* [69]; Świderski *et al.* [82]^c^
*Dollfusiella* sp. sensu	Spiral valve	Moreton Bay Q. Aus	Beveridge and Schaeffner [79]
*Parachristianella baverstocki*	Spiral valve	Sulu Sea off Kampung Tetabuan, Sabah, Malaysian Borneo	Schaeffner and Beveridge [83]^d^
*Parachristianella indonesiensis*	Spiral valve	Sulu Sea off Kampung Tetabuan, Sabah, Malaysian Borneo	Schaeffner and Beveridge [83]^d^
*Parachristianella monomegacantha*	Spiral valve	Sulu Sea off Kampung Tetabuan, Sabah, Malaysian Borneo	Schaeffner and Beveridge [83]^d^
	Spiral valve	Moreton Bay Q. Aus	Beveridge and Schaeffner [79]
*Prochristianella butlerae*	Spiral valve	Moreton Bay Q. Aus	Beveridge and Schaeffner [79]
*Prochristianella clarkeae*	Spiral valve	Moreton Bay Q. Aus	Beveridge and Schaeffner [79]
Platyhelminthes: Cestoda: Trypanorhyncha: Otobothriidae			
*Proemotobothrium southwelli*	Spiral valve	Moreton Bay Q. Aus	Beveridge and Schaeffner [79]
Platyhelminthes: Monopisthocotyla: Monocotylidea: Microbothriidae			
Dermopristis cairae* = *Dermophthirius* sp. of Perkins *et al.* (2009)	Skin, possibly nasal fossae	Coral Sea: *circa* Cairns	Kearn *et al.* [84]; Perkins *et al.* [85]; Whittington and Kearn [38]
Platyhelminthes: Monopisthocotyla: Monocotylidea: Monocotylidae			
*Calicotyle australis*	Cloaca, rectum, vent	Moreton Bay Q. Aus	Whittington *et al.* [86]^2^
*Mycteronastes icopae* = *Merizocotyle icopae*	Nasal Fossae/olfactory sacs	Moreton Bay Q. Aus	Cribb *et al.* [87]^c^; Kritsky *et al.* [88]; Mollaret *et al.* [89]^c^; Whittington *et al.* [90]^c^
	Nasal fossae	Coral Sea: Heron Is. Q. Aus	Beverley-Burton and Williams [91]^b^; Chisholm [92]^c^; Chisholm and Whittington [93–97]^c^; Chisholm *et al.* [98]^c^; Cribb *et al.* [87, 99]^c^; Hamwood *et al.* [100]^c^; Whittington *et al.* [90]^c^
	Nasal fossae	Makassar Strait, Muara Pasir, East Kalimantan, Indonesia	Chisholm and Whittington [101]
Neoheterocotyle rhinobatidis*	Gills	Moreton Bay Q. Aus	Kearn [102]^b^
	Gills	Coral Sea: Heron Is. Q. Aus	Chisholm [92]^c^; Chisholm and Whittington [93, 95, 96, 103]^c^, Chisholm *et al.* [98]^c^; Cribb *et al.* [104]^c^; Hamwood *et al.* [100]^c^; Mollaret *et al.* [89]^c^; Watson [105]^c^
*Neoheterocotyle rhynchobatis*	Gills	Moreton Bay Q. Aus	Kritsky and Chisholm [106];
	Gills	Coral Sea: Heron Is. Q. Aus	Chisholm and Whittington [96, 103]^c^
*Troglocephalus rhinobatidis* (Dasybatotreminae)	Gills	Moreton Bay Q. Aus	Kearn [102]^b^; Kritsky and Chisholm [106]
	Gills	Coral Sea: Heron Is. Q. Aus	Beverley-Burton [91]^b^; Chisholm [92]^c^; Chisholm and Whittington [93, 96]^c^; Chisholm *et al.* [98]^c^; Cribb *et al.* [104]^c^; Hamwood *et al.* [100]^c^; Kearn [102]^b^; Kritsky and Chisholm [106]; Mollaret *et al.* [89]^c^; Watson [105]^c^; Young [107]^c^
Platyhelminthes: Trematoda: Schistosomatoidea: Chimaerohemecidae			
Ogawaia glaucostegi* = *Myliobaticola* sp.	Heart	Moreton Bay	Cribb *et al.* [108]; Cutmore *et al.* [109]

*Glaucostegus typus* only known definitive host denoted by an asterisk

^a^Considered a *species inquirenda* by Caira *et al.* [110]

^b^Host reported as junior subjective synonym (*Rhinobatos batillum*)

^c^Host reported as junior subjective synonym *Rhinobatos typus* or *Rhinobatus typus*

^d^Host reported as *G*. *typus *sensu Naylor *et al.* [26]

^e^Host reported as junior subjective synonym *Rhinobatos armatus*

## Materials and Methods

### Host Sampling and Parasite Collection

**Fig. 1:**
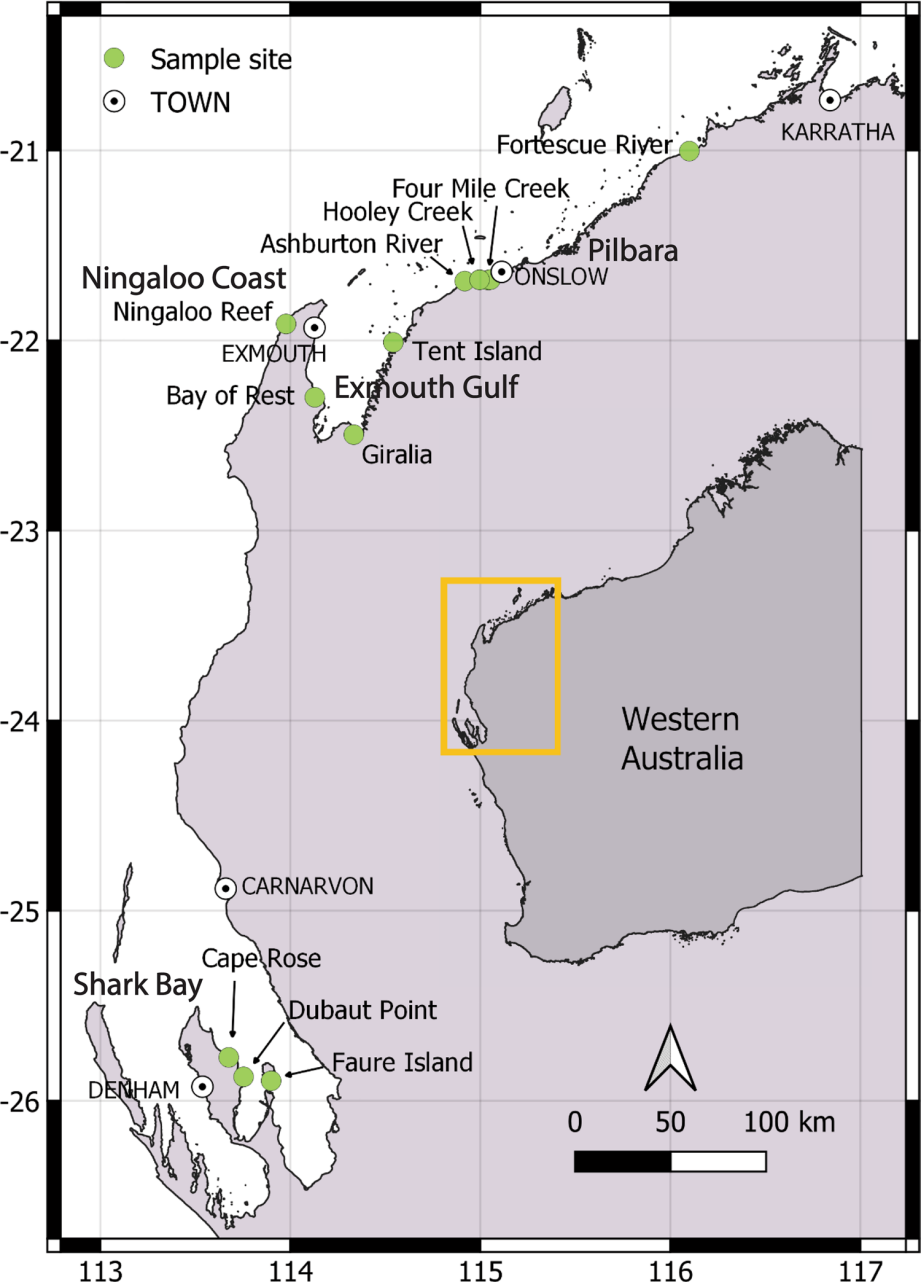
Sampling locations for the giant shovelnose ray, *Glaucostegus typus*, in Western Australia. Ashburton River includes samples from the Ashburton Delta/Hooley Lagoon

**Fig. 2:**
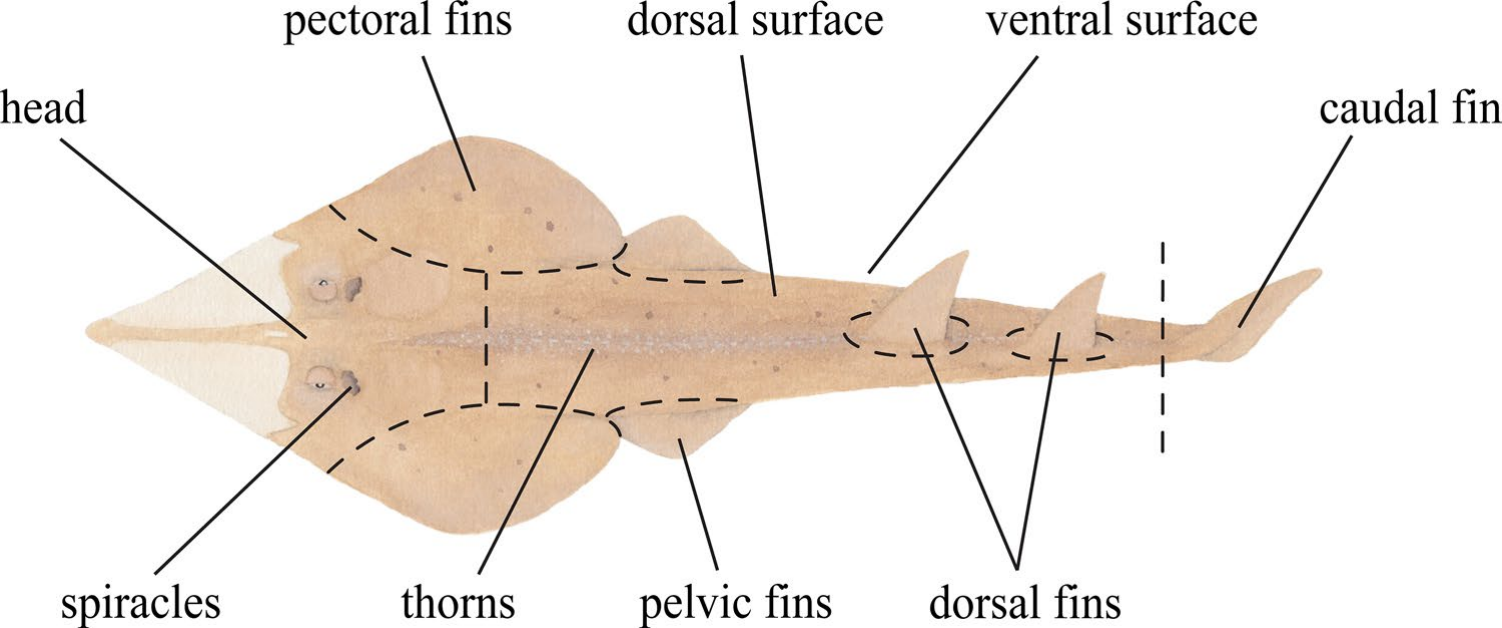
Gross morphology of the giant shovelnose ray, *Glaucostegus typus*, with infection site categories, excluding mouth and nares situated on the ventral surface (Illustration by Karissa Lear)

Targeted sampling for *G*. *typus* was conducted at multiple sites in four coastal regions in Western Australia (Fig. 1): the Pilbara region between October 2020 and August 2022; Exmouth Gulf between August 2020 and July 2022; Ningaloo Coast in February 2022; and Shark Bay between March and November 2022 (Table 2), which is considered the southern limit of the distribution of *G*. *typus* in the eastern Indian Ocean [16, 27–29]. Individual *G*. *typus* were caught using set nets, throw nets, encircled by monofilament gillnets in shallow waters, or by hand. Upon capture, rays were held on their backs in the extreme shallows with their gills submerged, inducing a state of tonic immobility. Stretched total length (TL; to the nearest mm) and sex were recorded. Subsequent estimates of age class were based on the clasper morphology of males (i.e., calcification) and growth data reported by White *et al.* [30]. Examinations for ectoparasites were conducted first on the ventral surface, after which the host was righted for examination of the dorsal surface, before being released. The gills, nasal lamellae and cloaca were not examined, because these areas could not be inspected non-invasively. Parasite attachment sites were recorded according to general body location (Fig. 2). Parasites were removed using forceps and most were immediately preserved in 100% ethanol, with some copepods and monopisthocotylan specimens fixed in 10% formalin for subsequent morphological study. Measurements of water temperature were recorded in each region using a YSI Professional Plus Multiparameter Meter (YSI Inc., Yellow Springs, United States of America) (Supplementary Table S1).

**Table 2:** Catch data for giant shovelnose rays, *Glaucostegus typus*, screened for ectoparasites in Western Australia

Site/region	Lat°	Long°	n	Sex		TL (mm)	Age class			Capture date
				F	M		0 +	≥ 1 +	Mt	(month/year)
FR/PR	− 21.009	116.098	4	2	2	795–1742	0	4	0	11/2021–08/2022
AR/PR	− 21.693	114.967	16	9	7	432–669	12	4	0	10/2020–04/2021
NR/NC	− 21.970	113.941	1	1	0	2205	0	0	1	02/2022
TI/EG	− 22.051	114.527	4	1	3	490–1186	2	2	0	12/2021
BR/EG	− 22.329	114.117	78	35	43	369–2092	6	70	2	09/2021–07/2022
GS/EG	− 22.505	114.341	17	8	9	485–778	5	12	0	08/2020
CR/SB	− 25.747	113.663	5	3	2	1740–1910	0	3	2	11/2022
DP/SB	− 25.998	113.742	34	13	21	390–2073	10	20	4	03/2022–11/2022
FI/SB	− 25.860	113.852	27	12	15	384–2107	6	16	5	03/2021

Fortescue River and Ashburton River includes samples from adjacent tidal creeks. Age classes based on White *et al.* [35]

Abbreviations: AR, Ashburton River; BR, Bay of Rest; CR, Cape Rose; DP, Dubaut Point; EG, Exmouth Gulf; FI, Faure Island; FR, Fortescue River; GS, Giralia Station; NC, Ningaloo Coast; NR, Ningaloo Reef; PR, Pilbara Region; SB, Shark Bay; TI, Tent Island; Lat, latitude; Long, longitude; F, female; M, male; n, number of host individuals examined; TL, total length; 0 + , young of the year; ≥ 1 +, juveniles that are one year and older; Mt, mature

### Morphological Study

Parasite identifications were based solely on morphology. For mounting of select copepods and monopisthocotylans, specimens initially preserved in formalin were later transferred to absolute ethanol using a graded ethanol series: 40, 60, 75 and twice at 100% for approximately 1 h per stage. Copepod specimens were cleared in lactophenol and mounted (unstained) in Canada balsam. Monopisthocotylans were treated as described in Ingelbrecht *et al.* [31, 32]. Leeches and isopods were examined as uncleared and unstained wet mounts in absolute ethanol. Slide-mounted specimens were examined and photographed using an Olympus BX50 compound microscope, with Nomarski interference contrast, fitted with an Olympus DP71 digital microscope camera and U-CMAD3 adaptor (Olympus Inc., Tokyo, Japan). Wet mounts were examined and photographed using an Olympus SZX7 stereo microscope with an Olympus DF PLAPO auxiliary lens. Select material has been deposited with the Crustacea and Worms collection of the Western Australian Museum (WAM).

### Data Analyses

Prevalence (proportion of infected hosts), intensity of infection (mean number of parasites per infected host) and parasite abundance (equivalent to prevalence × intensity) were calculated for each ectoparasite species using the online tool QPweb v.1.0.15 [33]. Bias-corrected and accelerated bootstrap 95% confidence intervals were calculated for mean infection intensity. For ectoparasite species with adequate sample sizes (i.e., present on ≥ 10 host individuals), aggregation was investigated in QPweb from variance/mean ratios (*s*^*2*^*/m*) and negative binomial exponent values (*k*), with aggregation indices calculated across all screened *G*. *typus*.

Predictors of parasite abundance were investigated for ectoparasite species with adequate sample sizes, using a series of models in R v.4.2.3 [34]. Because parasite abundances are aggregated by nature, two distributions were compared to account for overdispersion: zero-inflated negative binomial and zero-inflated Poisson. These models assume that excess zeros are generated by a separate process from count data and are therefore modelled separately, with the first modelled distribution relating to the odds of infection for a host (i.e. whether it is infected or not) and the second relating to the intensity of infection. Fixed predictors of abundance incorporated into initial models included host TL and sex, sampling season and region. Models using all combinations of predictor variables and their interactions were created. Samples from the Ningaloo Coast were not included due to inadequate sample size (i.e., < 10 screened *G*. *typus*). The best-fit model from this set was chosen through examination of Akaike’s Information Criterion (AIC), using the lowest AIC score, or, if the lowest AIC scores were within two units, the model with the fewest degrees of freedom (DF) was selected.

## Results

A total of 186 *G*. *typus* (84 females, 102 males) were examined for ectoparasites (Table 2). Ectoparasites were detected on 98 *G*. *typus*, of which 9% were estimated to be young of the year (YOY) (< 520 mm TL), 80% were juveniles older than one year (523–1880 mm TL) and 11% were mature (> 1880 mm TL).

### Parasite Taxa

Five ectoparasite taxa were detected on *G*. *typus* (Table 3): *Caligus furcisetifer* Redkar, Rangnekar & Murti, 1949 (Copepoda: Caligidae), *Dermopristis cairae* Whittington & Kearn, 2011 (Monopisthocotyla: Microbothriidae), *Branchellion plicobranchus* Sanjeeva Raj, 1953 and *Stibarobdella macrothela* (Schmarda, 1861) (Hirudinida: Piscicolidae), and praniza larvae of one or more unidentified gnathiid isopod species (Isopoda: Gnathiidae). Seventeen parasite specimens from *G*. *typus* were deposited as vouchers: four *C*. *furcisetifer* (WAM C84062–C84065), four *D*. *cairae* (WAM V12775–V12778), one *B*. *plicobranchus* (WAM V12770), four *S*. *macrothela* (WAM V12771–V12774) and four gnathiid praniza larvae (WAM C84058–C84061).

**Table 3:** Summary of ectoparasites infecting the giant shovelnose ray, *Glaucostegus typus* (n = 186), in Western Australia

Parasite	Prevalence	Intensity	Infection site	Region
Caligidae				
*Caligus furcisetifer*	31% (57)	1–20 (3.4; 2.6–4.5)	cf; df; ds; hd; pc; pv; vs	EG; NC; PR; SB
Gnathiidae				
Gnathiids	13% (24)	1–19 (3.5; 2.2–5.8)	hd, nrs, sp	EG; NC; SB
Microbothriidae				
*Dermopristis cairae*	2% (3)	1–29 (12.7; 1–22)	df; ds (mid-line)	SB
Piscicolidae				
*Branchellion plicobranchus*	1% (1)	1	hd	NC
*Stibarobdella macrothela*	40% (74)	1–50 (6.0; 4.7–8.7)	bc; cf; ds; hd; pc; pv; vs	EG; PR; SB

Prevalence is the percentage of hosts infected, followed by the number of hosts infected in parentheses. Intensity is the range of intensity of infection, followed by the mean and 95% confidence intervals in parentheses. Abbreviations: EG, Exmouth Gulf; NC, Ningaloo Coast; PR, Pilbara region; SB, Shark Bay; bc, buccal cavity; cf, caudal fin (including peduncle); df, dorsal fins; ds, dorsal body surface not in proximity to fins; hd, head (excluding buccal cavity, nares and spiracles); nrs, nares; pc, pectoral fins; pv, pelvic fins; sp, spiracles; vs, ventral body surface not in proximity to fins

**Fig. 3:**
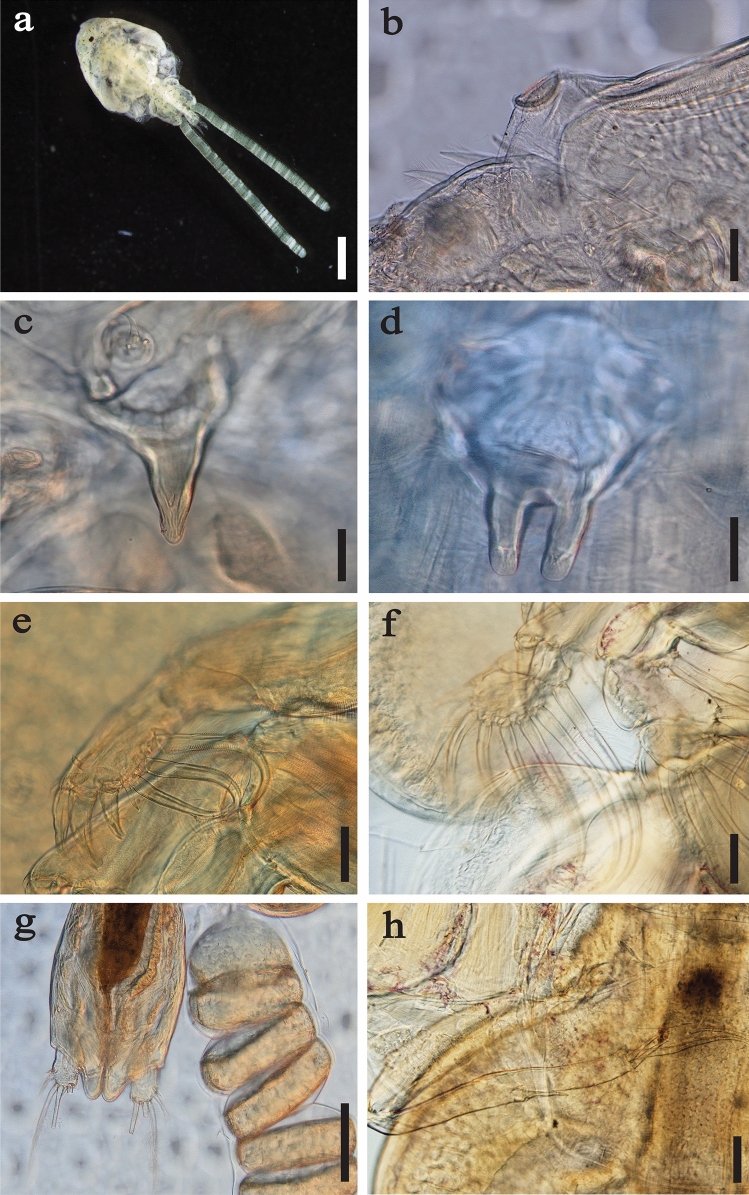
*Caligus furcisetifer* from the giant shovelnose ray, *Glaucostegus typus*, in Western Australia. **a** Habitus, dorsal view. **b** Right lunule and side of frontal plate, ventral view. **c** Right maxillule, ventral view. **d** Sternal furca, ventral view. **e** Right leg 1 exopod, ventral view. **f** Right leg 2 exopod, ventral view. **g** Abdomen with caudal rami and egg sacs, ventral view. **h** Right leg 4 exopod, ventral view. Scale bars: a = 1 mm; b–e = 50 μm; g, h = 100 μm

*Caligus furcisetifer* (Fig. 3) were identified on the basis of body size of specimens (4.2–4.7 mm body length; n = 3); rounded cephalothorax, approximately 1.1 times longer than wide, with maximum width approximately half of the distance from the anterior end, comprising approximately 63% of total body length (Fig. 3a); minute lunules, shifted laterally on the frontal plate (Fig. 3b); presence of a small, triangular sclerite on the ventral surface that projects distally over maxillule dentiform process base; genital complex with rounded corners, approximately 1.3 times wider than long (Fig. 3c); sternal furca with widely spaced, slightly divergent tines (Fig. 3d); presence of an accessory process on middle and inner terminal spines and reduced apical seta on the terminal exopodal segment of leg 1 (Fig. 3e); and subequal middle and outer spines on the terminal exopodal segment of leg 4 (Fig. 3h) [35–37]. *Caligus furcisetifer* was encountered in all sampled regions (Table 3) and on all exterior surfaces of the host body (i.e., excluding the nares, spiracles and buccal cavity), but were predominantly encounterd on the head, which accounted for 60% of the combined total infections by this species (Table 4).

**Table 4:** Summary of infection site data for ectoparasites encountered on ≥ 10 giant shovelnose rays, *Glaucostegus typus*, in Western Australia

Microhabitat	*Caligus furcisetifer*			Gnathiids			*Stibarobdella macrothela*		
	CTI	SI	Prv	CTI	SI	Prv	CTI	SI	Prv
Head	88	2.7	73%	14	3.5	17%	47	2.1	36%
Mouth	–	–	–	–	–	–	72	3.0	39%
Nares	–	–	–	50	2.5	87%	256	5.1	82%
Spiracles	–	–	–	10	1.4	30%	9	4.5	3%
Dorsal fins	16	2.3	16%	–	–	–	–	–	–
Pectoral fins	11	1.0	24%	–	–	–	10	1.3	10%
Pelvic fins	12	2	13%	–	–	–	4	1.0	7%
Caudal fin	8	1.6	11%	–	–	–	1	1.0	2%
Dorsal surface	3	1.0	7%	–	–	–	1	1.0	2%
Ventral surface	8	1.6	11%	–	–	–	5	1.0	8%

Combined total infections (CTI) is the sum total of infections of each taxon across all screened *G. typus.* Mean microhabitat site intensity (SI) and prevalence (Prv) of each taxon is calculated for each microhabitat out of the total number of *G*. *typus* infected with at least one *Caligus furcisetifer* (n = 57), gnathiids (n = 24), or *Stibarobdella macrothela* (n = 74)

**Fig. 4:**
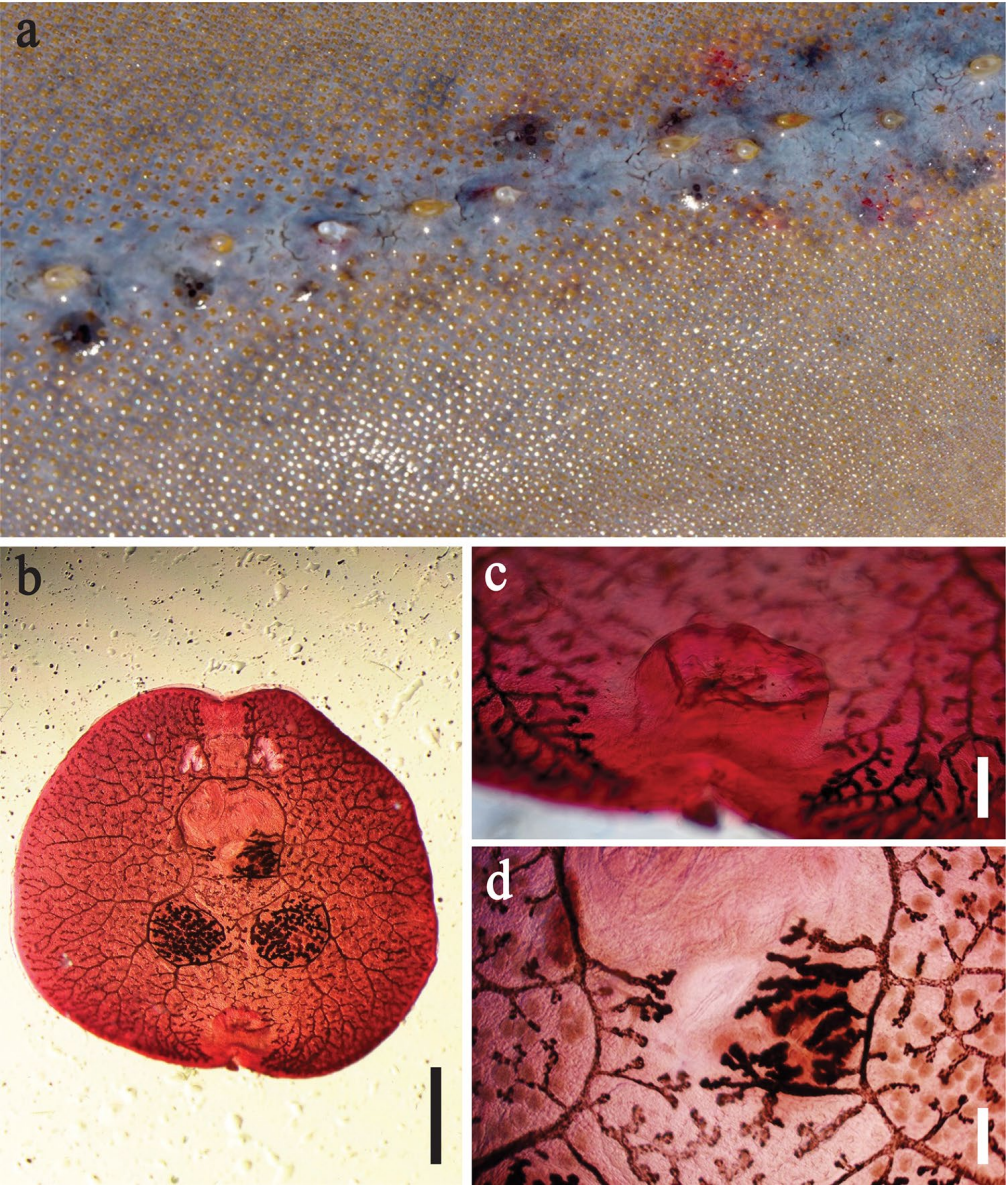
*Dermopristis cairae* from the giant shovelnose ray, *Glaucostegus typus*, in Western Australia. **a** In situ adjacent to the series of thorns along the dorsal mid-line (Photograph by Leon Deschamps). **b** Habitus, ventral view. **c** Simple haptor, ventral view. **d** Fine details of gut diverticula and oötype, posterior to tubular male reproductive tract, ventral view. Scale bars: b = 1 mm; c, d = 200 μm

*Dermopristis cairae* (Fig. 4) was identified on the basis of body size and shape of specimens (4.6–5.3 mm body length, 4.9–5.5 mm body width; n = 3); absence of transverse ridges on the ventral surface (Fig. 4b); absence of a seminal receptacle; simple, hookless haptor (Fig. 4c); and fine details of gut diverticula adjacent to the vas deferens and the oötype (Fig. 4d) [38]. All specimens were found on the dorsal surface of *G*. *typus* (Table 4), occurring almost exclusively adjacent to the series of thorns running along the mid-line (Fig. 4a), and were encountered only at Shark Bay (Table 3).

**Fig. 5:**
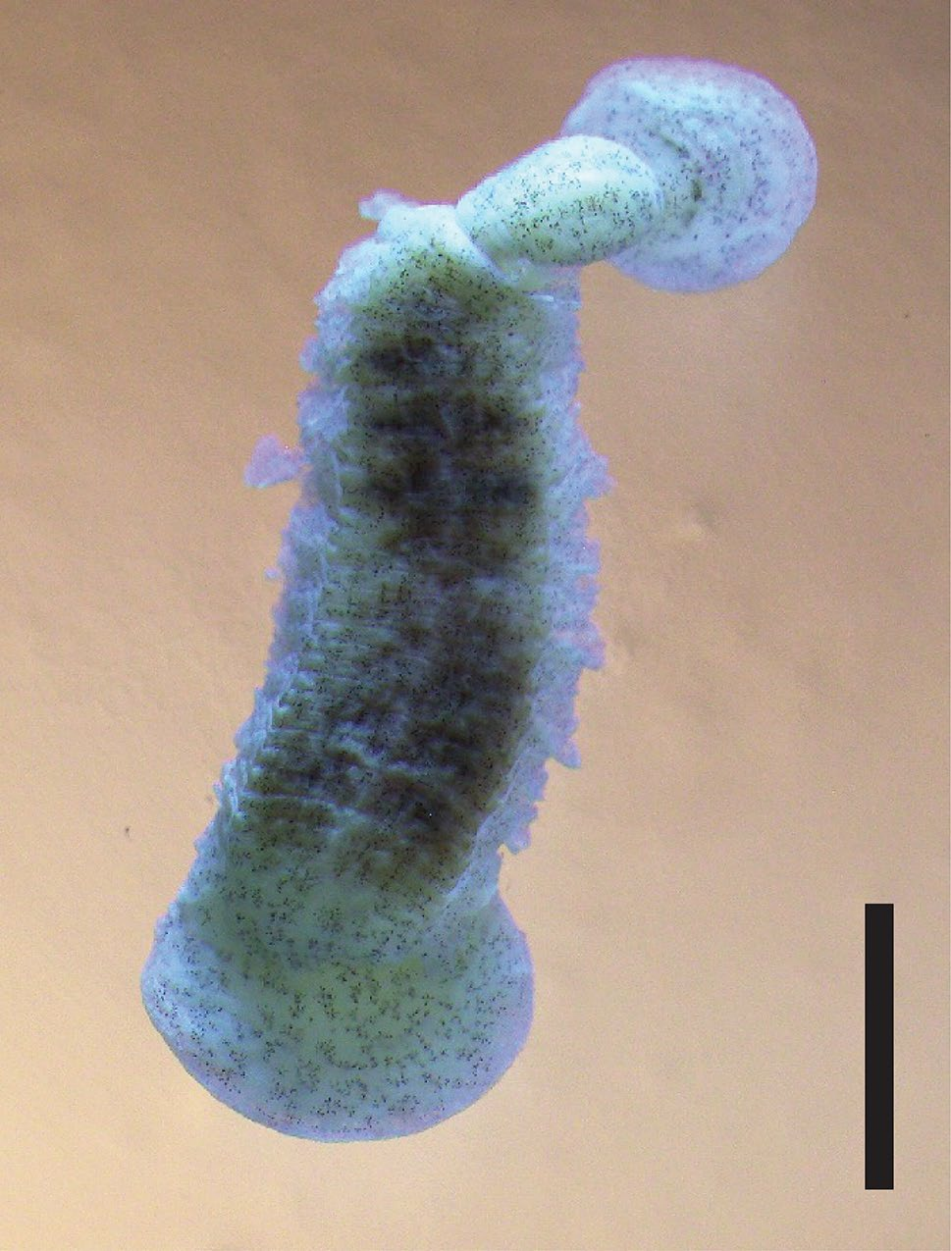
Immature *Branchellion plicobranchus*, dorsal view, from the giant shovelnose ray, *Glaucostegus typus*, in Western Australia. Scale bar = 2 mm

Only a single, immature specimen of *B*. *plicobranchus* was encountered, which was identified based on the 33 pairs of leaf-like branchiae (Fig. 5); absence of eyespots on the oral sucker; and absence of an obvious bilobed hump on the ventral surface of segment VII [39]. This specimen was recovered from the head of a *G*. *typus* on the Ningaloo Coast (Table 3).

**Fig. 6:**
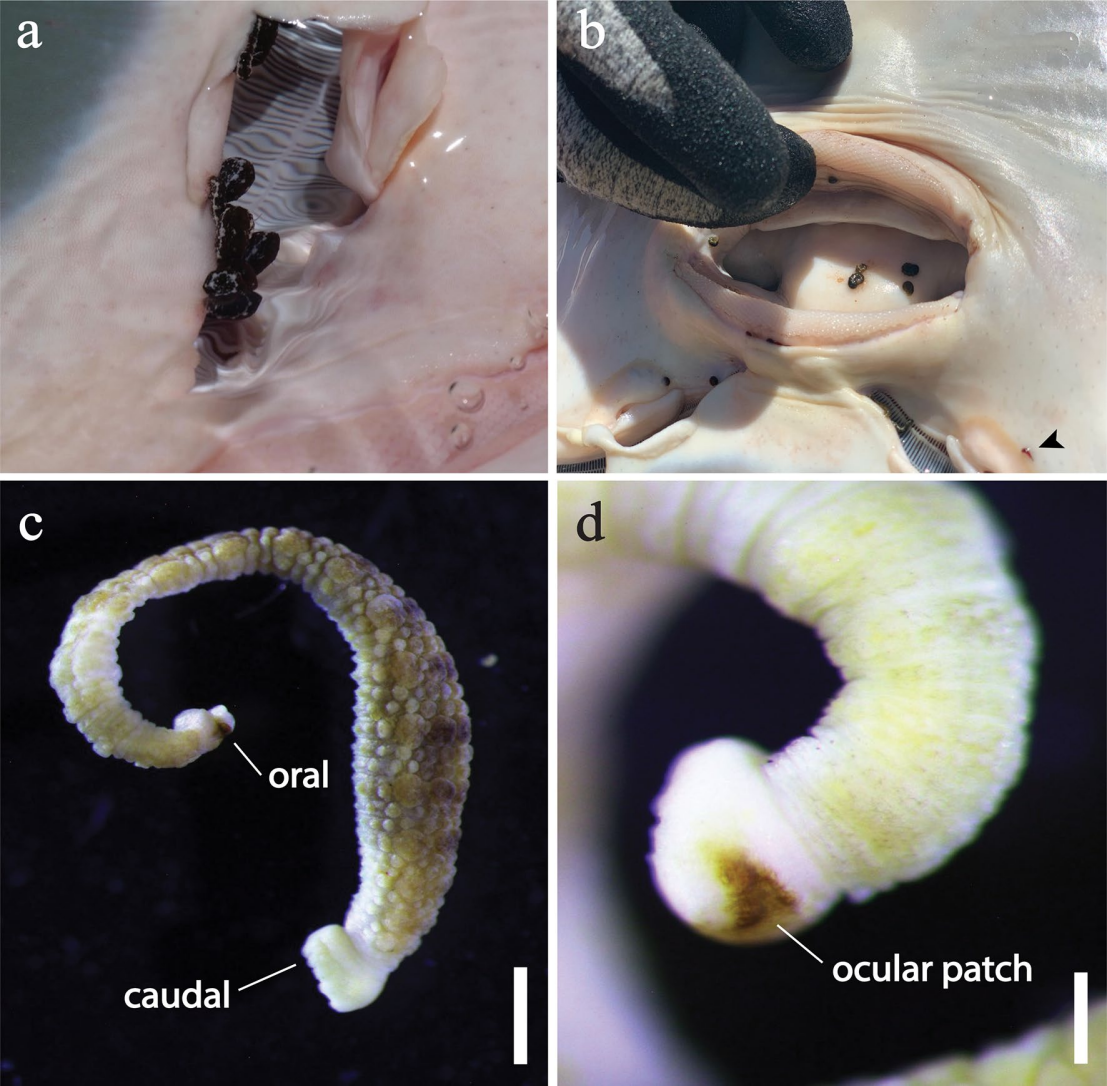
*Stibarobdella macrothela* from giant shovelnose ray, *Glaucostegus typus*, in Western Australia. **a** In situ in the naris of a *G*. *typus* in Shark Bay, with predominantly black pigmentation (Photograph by Leon Deschamps). **b** In situ in the buccal cavity and, or adjacent to, the nares of *G*. *typus* in the Pilbara region, including with dark red pigmentation (bottom right) (Photograph by David Morgan). **c** Habitus, lateral view. **d** Oral sucker, lateral view, with ocular patch. Abbreviations: caudal, caudal sucker; oral, oral sucker. Scale bars: c = 1 mm; d = 200 μm (colour figure online)

*Stibarobdella macrothela* (Fig. 6) was identified based on the large, wart-like tubercles present both dorsally and ventrally on the annuli of each trachelosome and urosome segment of specimens (Fig. 6c); two large, trumpet shaped ocular patches on the oral sucker (Fig. 6d); and large caudal sucker [40, 41]. Additionally, two new variations in pigmentation were observed on specimens in situ: predominantly black with some white streaks occurring laterally along the length of the urosome (Fig. 6a), and uniformly dark red; the latter of which was observed infrequently and only for immature specimens (Fig. 6b). Most *S*. *macrothela* were found on the head, particularly proximal to the nares (63%) and buccal cavity (18%), which were occasionally heavily infected (Table 4). *Stibarobdella macrothela* was encountered in all regions except for the Ningaloo Coast (Table 3).

**Fig. 7:**
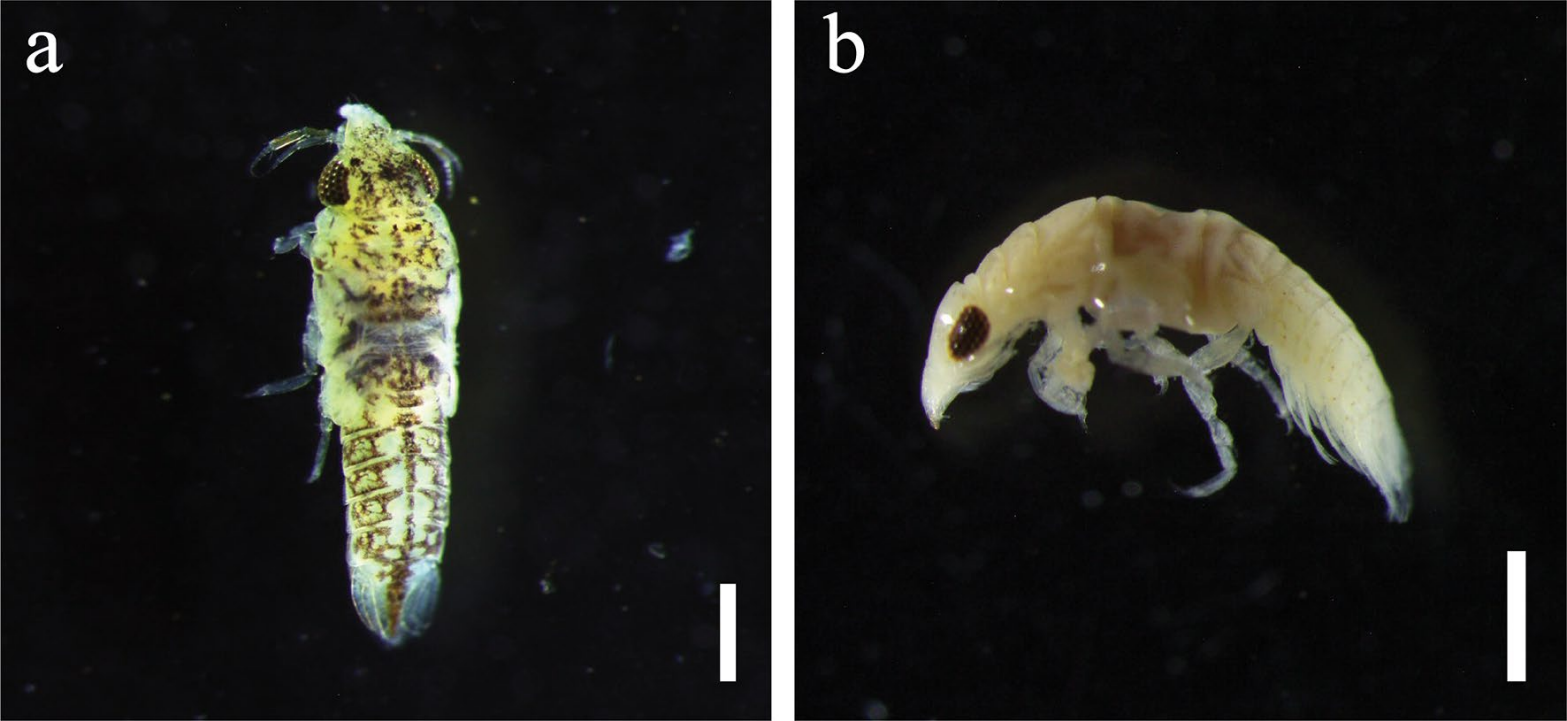
Gnathiids from the nares of the giant shovelnose ray, *Glaucostegus typus*, in Western Australia. **a** Habitus, dorsal view, with predominantly yellow pigmentation. **b** Habitus, lateral view, with predominantly white pigmentation. Scale bars = 500 μm (colour figure online)

Gnathiid taxonomy is typically based on the morphology of adult males [42]; therefore, no attempt has been made to determine the specific identity of recovered pranizae. Few species descriptions of gnathiids include pigmentation as a characteristic [43], and colouration often disappears through fixation [44]. Nevertheless, several distinct body pigmentations were noted in situ on collected pranizae during this study: predominantly yellow, with occasional uniform, brown streaks on the cephalosome, pereon and pleon (Fig. 7a); as well as predominantly white pigment, with sparse, light spots on the pleon (Fig. 7b). Gnathiids were encountered in all regions except for the Pilbara (Table 3), and exclusively on the heads of rays, with most infections (68%) occurring in the nares (Table 4).

### Aggregation, Presence and Intensity

**Fig. 8:**
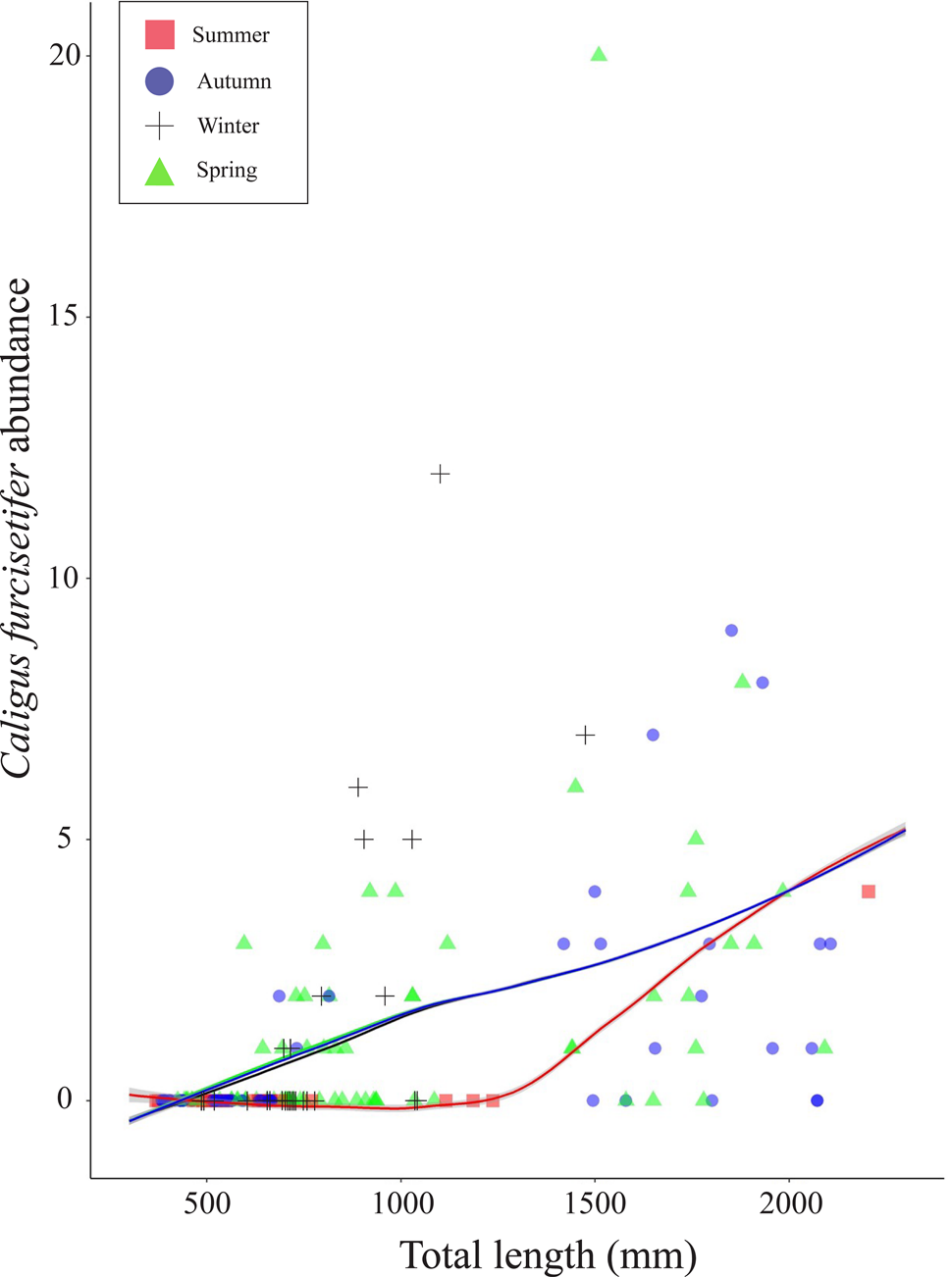
Sampled (data points) and model-predicted (curve) abundance of *Caligus furcisetifer*, infecting the giant shovelnose ray, *Glaucostegus typus* (n = 185), in Western Australia. Abundance is displayed relative to host total length and sampling season. Standard error margins for model-predicted intensity are denoted by grey shading either side of curve. Sample from the Ningaloo Coast not included

*Caligus furcisetifer, S*. *macrothela* and gnathiids were each highly aggregated (*s*^*2*^*/m* = 5.51, 13.52, 8.02 and *k* = 0.19, 0.20, 0.08, respectively). The AIC values indicated a zero-inflated negative binomial model was the best fit for all three taxa (see Supplementary Table S2). For *C*. *furcisetifer*, the best fit model included host TL and season as predictors of infection presence (AIC = 377.29, DF = 8), with odds of infection increasing with host TL and considerably lower in summer (Fig. 8) (Supplementary Table S3). Host TL was maintained in the best-fit model as the sole predictor of *C*. *furcisetifer* infection intensity, which increased with TL, especially on host individuals > 1300 mm in length (Fig. 8) (Supplementary Table S4).

**Fig. 9:**
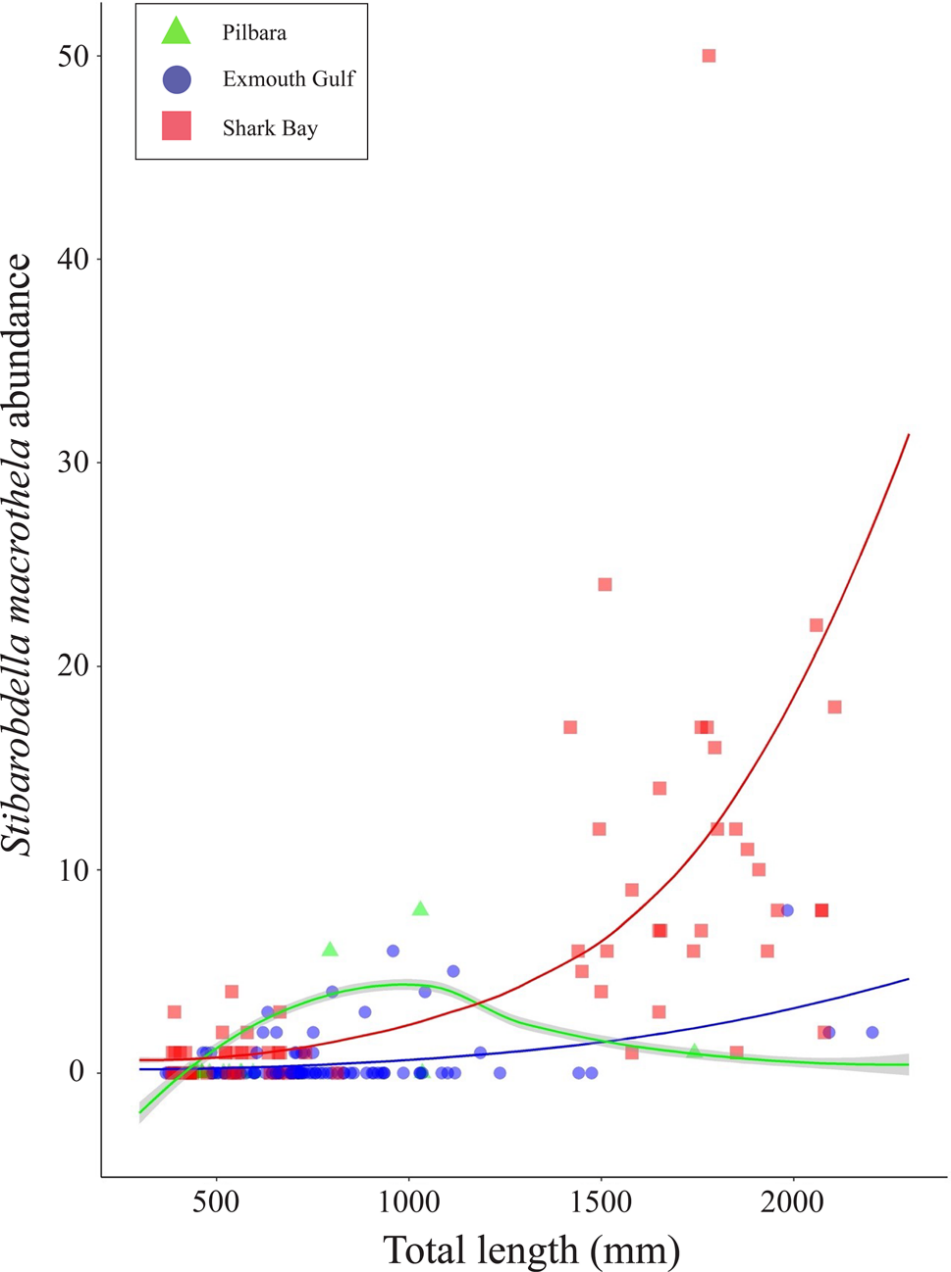
Sampled (data points) and model-predicted (curve) abundance of *Stibarobdella macrothela* infecting the giant shovelnose ray, *Glaucostegus typus* (n = 185), in Western Australia. Abundance is displayed relative to host total length and sampling region. Standard error margins for model-predicted intensity are denoted by grey shading either side of curve. Sample from the Ningaloo Coast not included

For *S*. *macrothela*, host TL and sample region, and their interaction, were in the best-fit model as predictors of infection presence and intensity (AIC = 511.32, DF = 13) (Supplementary Table S3, S4). Odds and intensity of infection increased with host TL, especially on host individuals > 1500 mm TL, and were considerably higher in Shark Bay compared to other regions (Fig. 9).

**Fig. 10:**
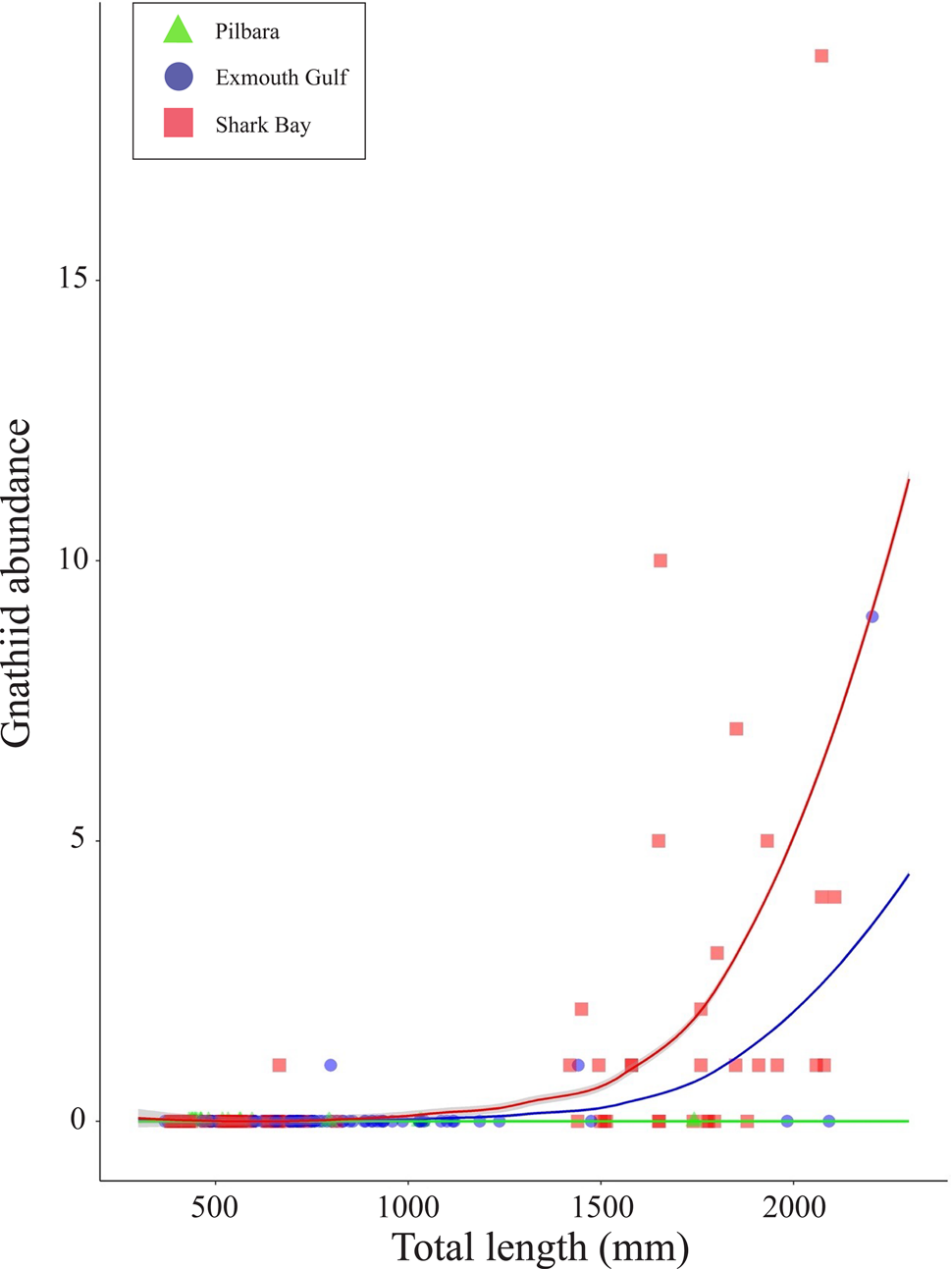
Sampled (data points) and model-predicted (curve) abundance of gnathiid praniza larvae infecting the giant shovelnose ray, *Glaucostegus typus* (n = 185), in Western Australia. Abundance is displayed relative to host total length and sampling region. Standard error margins for model-predicted intensity are denoted by grey shading either side of curve. Sample from the Ningaloo Coast not included

For gnathiids, the best-fit model had sample region as the sole predictor of infection presence (AIC = 165.83, DF = 6) (Supplementary Table S3), with odds of infection greatest in Shark Bay. Host TL was the sole predictor of gnathiid infection intensity (Supplementary Table S4), which increased with host TL, especially on host individuals > 1500 mm TL (Fig. 10).

## Discussion

This is the first study to investigate parasites exploiting the Critically Endangered giant shovelnose ray, *Glaucostegus typus*, in Western Australia. These are the first records of *B*. *plicobranchus* and *S*. *macrothela* infecting *G*. *typus*, although Burreson [40] speculated that the former likely infects this host. This research extends the geographical range of *B*. *plicobranchus* and *C*. *furcisetifer* south in the eastern Indian Ocean to the Ningaloo Coast and Shark Bay, respectively. *Dermopristis cairae* was originally described based on specimens collected during freshwater bathing treatment of *G*. *typus* at Cairns Marine Aquarium Supply in Queensland, Australia, with ambiguity pertaining to its microhabitat [38], which this study clarifies (occurring mostly along the thorn ridge on the dorsal midline). The microhabitat of *D*. *cairae* is distinct from its congeners; *Dermopristis pterophila* Ingelbrecht, Morgan & Martin, 2022, exhibits affinity for the pectoral and pelvic fin bases of the green sawfish, *Pristis zijsron* Bleeker, 1851 [32], and *Dermopristis paradoxa* Kearn, Whittington & Evans-Gowing, 2010, occurs proximal to the mouth of the largetooth sawfish, *Pristis pristis* (Linnaeus, 1758) [45].

In Western Australia alone, *C*. *furcisetifer* is known to infect *P*. *pristis*, *P*. *zijsron* and the eyebrow wedgefish, *Rhynchobatus palpebratus* Compagno & Last, 2008, whereas *B*. *plicobranchus* has been encountered on *P*. *zijsron*, and *S*. *macrothela* has been encountered on *P*. *zijsron*, as well as the sandbar shark, *Carcharinus plumbeus* (Nardo, 1827), the blacktip reef shark, *Carcharhinus melanopterus* (Quoy & Gaimard, 1824), and an unidentified wobbegong (Orectolobidae) [31, 36, 40].

### Seasonal and Regional Variations in Infections

In our analyses, the best-fit models demonstrated that *G. typus* were less likely to be infected by *C. furcisetifer* during summer. Seasonal fluctuations of *C*. *furcisetifer* prevalence are possibly related to variations in copepod recruitment or host behaviour that affect the probability of parasite transmission [46, 47]. The activity space of *G*. *typus* is known to increase significantly during winter, likely in response to a decrease in resource availability, which may increase the probability of contact with the infectious copepodid stage of *C*. *furcisetifer* [48]. Conversely, habitat use is confined to smaller areas throughout the warmer months of the year, particularly in summer, which may limit contact with copepodids [48]. An alternative, but not mutually exclusive, explanation for this seasonal pattern in prevalence is a lack of immigration of infected *G. typus* into the study area in summer when activity is relatively low [48]. It seems unlikely that seasonal variation in prevalence of *C*. *furcisetifer* is directly related to water temperature, because temperatures recorded in autumn and summer were similar (mean = 26.5 ± 0.1 °C and 27.3 ± 0.2 °C, respectively) and this species is known to occur on *P*. *pristis* in the Fitzroy River estuary, Western Australia, and the Leichhardt River estuary, Queensland, where water temperatures occasionally exceed 30 °C [36, 49], although seasonal differences in parasite abundance can vary among host species [50]. Therefore, additional work is needed to understand the driver of the seasonal fluctuation in *C*. *furcisetifer* prevalence.

Regional variations among parasite populations are common, with differences in parasite diversity and abundance typically increasing with geographical distance [51–53]. For *S*. *macrothela* and gnathiids, likelihoods of infection were greatest in Shark Bay, with *S*. *macrothela* intensity of infection also greatest in this region. In Australian waters, previous encounters with *S*. *macrothela* have occurred mostly north of the Tropic of Capricorn [40], suggesting it is perhaps a predominantly tropical species. It is therefore intriguing that the likelihood and intensity of infections by *S*. *macrothela* were greatest in Shark Bay, which signifies a transition zone between tropical and temperate conditions, although this species has previously been detected as far south as Point Peron, almost 700 km south of Shark Bay [40]. These results suggest that *S*. *macrothela* could in fact be most suited for subtropical conditions and that previous records simply reflect where this species has been recorded, rather than where it is most abundant. The differences in infections of *S*. *macrothela* between the Pilbara, Exmouth Gulf and Shark Bay may also be a consequence of biotic or abiotic conditions that vary between regions, such as salinity or turbidity. However, additional work is required to determine this. Considering the presence of gnathiids also correlated positively with latitude, it is highly plausible that the gnathiid pranizae encountered during this study are most suited for subtropical or temperate conditions.

### Importance of Host Size

Our analyses demonstrated that larger *G*. *typus* are more likely to be infected by *C*. *furcisetifer* and *S*. *macrothela*, and carry a greater intensity of infection of these species, as well as gnathiids, across the study area. Correlations between host size and parasite intensity are common in wild populations; larger, older hosts provide greater surface area for parasites to colonise, have had more time to accumulate parasites and often have home ranges that expand with growth, which may increase their exposure to parasites [54–56, 62]. Such trends have been reported for numerous species, including for *C*. *furcisetifer* on *P. zijsron* in Western Australia, where copepod abundance was found to increase exponentially with host TL, as well as for gnathiid pranizae on several labrid species in Queensland, Australia [31, 58, 59]. Like *P*. *zijsron*, the activity space of *G*. *typus* is known to increase with age, with mature individuals also utilising a greater variety of habitats than juveniles, likely increasing their exposure to parasites [60, 61]. As such, these data suggest that as host individuals grow, they may increasingly contribute to the spread and proliferation of parasites including *C*. *furcisetifer*, *S*. *macrothela* and gnathiid pranizae within and among populations.

## Conclusion

Of the ectoparasites encountered during this study, *D. cariae* is the only species considered to be specific to *G*. *typus*. Collectively, species of *Dermopristis* are known only from rhinopristiform fishes in Australian waters, and *D*. *cairae* has been previously reported only from *G*. *typus* within captive settings [38, 45]. Modifications to the classification criteria for the IUCN Red List of Threatened Species have been proposed to classify parasites via referencing the threatened status of their host/s [5, 62]. However, this could result in an underestimation of the true extinction risk faced by parasites, due to the over-dispersed nature of infections, particularly species that are host-specific [5, 63]. Considering that *D*. *cairae*, which is presumably restricted to *G*. *typus*, was encountered at a very low prevalence (2%) and in only a single locality, these findings suggest that the threat of extinction faced by *D*. *cairae* is likely greater than that of its host, which is considered to be a species of Least Concern within Australian waters and Critically Endangered globally [14, 24].

## Supplementary Information

Below is the link to the electronic supplementary material.

Supplementary file1 (DOCX 32 KB)

## Data Availability

The data supporting the findings of this study are available from the corresponding author upon reasonable request.
