# Supplementary material for: Ectoparasites of the Critically Endangered Giant Shovelnose Ray Glaucostegus typus in the Eastern Indian Ocean, with a Summary of the Known Metazoan Parasites
Source: Acta Parasitol. 2024 Sep 17;69(4):1937–54. doi: 10.1007/s11686-024-00918-8 (PMC11649752; doi:10.1007/s11686-024-00918-8)
Supplement: Supplementary file 1 — Supplementary file1 (DOCX 32 KB) [file 11686_2024_918_MOESM1_ESM.docx]

**Supplementary Table S1** Summary of water temperature (°C) measurements collected from sample sites in the Exmouth Gulf, Ningaloo Coast, Pilbara and Shark Bay regions. Measurements were taken from the surface, mid-depth, and bottom of the water column at each site. Measurements are the range, followed by the mean ± standard error in parentheses.

| Region | Season | Month | Temperature (°C) |
| --- | --- | --- | --- |
| Exmouth Gulf | Summer | December, January | 26.3–28.1 (27.3 ± 0.2) |
|  | Winter | July, August | 22.6–25.3 (23.9 ± 0.2) |
|  | Spring | September, October, November | 23.4–25.3.0 (24.9 ± 0.1) |
| Ningaloo Coast | Summer | February | 27.3 |
| Pilbara | Autumn | April | 26.0 |
|  | Winter | August | 22.3 |
|  | Spring | October, November | 23.4–26.0 (24.2 ± 0.2) |
| Shark Bay | Autumn | March | 26.3–26.8 (26.6 ± 0.1) |
|  | Spring | November | 21.5–21.9 (21.6 ± 0.1) |

**Supplementary Table S2** Model types tested for best-fit in predicting the presence and intensity of Caligus furcisetifer, Stibarobdella macrothela and gnathiid pranizae on the giant shovelnose ray, Glaucostegus typus, in Western Australia. Predictor variables included in each model were host total length and sex, sample region and season.

| Parasite | Model | AIC | DF |
| --- | --- | --- | --- |
| Caligus furcisetifer | Zero-inflated negative binomial* | 383.36 | 17 |
|  | Zero-inflated Poisson | 423.49 | 16 |
| Stibarobdella macrothela | Zero-inflated negative binomial* | 524.85 | 17 |
|  | Zero-inflated Poisson | 633.95 | 16 |
| Gnathiid Pranizae | Zero-inflated negative binomial* | 166.58 | 17 |
|  | Zero-inflated Poisson | 192.13 | 16 |

The set of best-fit predictors for each parasite is denoted by an asterisk, based on the lowest Akaike’s Information Criterion (AIC) value, or, if lowest AIC values were within two units of each other, the set of predictors with the lowest degrees of freedom and within two units of lowest AIC value.

**Supplementary Table S3** Predictor variables tested for best-fit for predicting the presence of infection of Caligus furcisetifer, Stibarobdella macrothela and gnathiid pranizae on the giant shovelnose ray, Glaucostegus typus, in Western Australia. A zero-inflated, negative binomial distribution model was used for each species. A ‘+’ denotes an effect of multiple variables; a ‘×’ denotes an effect of multiple variables, including their interaction. Variables retained in models as best-fit predictors of infection intensity for each species are as follows: C. furcisetifer, host total length (TL); S. macrothela, TL, sample region and their interaction; gnathiid pranizae, TL.

| Parasite | Predictor | AIC | DF |
| --- | --- | --- | --- |
| *Caligus furcisetifer* | control | 419.36 | 4 |
|  | TL | 381.53 | 5 |
|  | sex  region | 421.47  423.49 | 5  6 |
|  | season | 418.35 | 7 |
|  | TL + sex  TL + region | 381.33  385.64 | 6  7 |
|  | TL + season*  sex + region | 377.29  424.52 | 8  7 |
|  | sex + season  region + season  TL + sex + region | 419.84  419.92  385.65 | 8  9  8 |
|  | TL + sex + season  TL + region + season  sex + region + season  TL + sex + region + season | 377.72  381.70  422.01  382.22 | 9  10  10  11 |
|  | TL × sex | 383.02 | 7 |
|  | TL × region  TL × season  sex × region | 383.65  378.76  428.91 | 9  11  9 |
|  | sex × season | 425.48 | 11 |
|  | TL × sex × region | 396.37 | 15 |
|  | TL × sex × season | 395.16 | 19 |
| *Stibarobdella macrothela* | control | 533.18 | 8 |
|  | TL | 534.42 | 9 |
|  | sex  region | 535.39  519.78 | 9  10 |
|  | season | 539.88 | 11 |
|  | TL + sex  TL + region | 534.66  513.67 | 10  11 |
|  | TL + season  sex + region | 532.98  521.46 | 12  11 |
|  | sex + season  region + season  TL + sex + region | 538.99  523.61  515.66 | 12  13  12 |
|  | TL + sex + season  TL + region + season  sex + region + season  TL + sex + region + season | 533.73  518.22  524.91  520.08 | 13  14  14  15 |
|  | TL × sex | 536.69 | 11 |
|  | TL × region*  TL × season  sex × region | 511.32  537.23  525.77 | 13  15  13 |
|  | sex × season | 543.01 | 15 |
|  | TL × sex × region | 524.91 | 19 |
|  | TL × sex × season | 544.21 | 23 |
| Gnathiid pranizae | control | 167.92 | 4 |
|  | TL | 168.58 | 8 |
|  | sex  region* | 170.03  165.83 | 5  6 |
|  | season | 167.86 | 7 |
|  | TL + sex  TL + region | 166.53  167.45 | 6  7 |
|  | TL + season  sex + region | 168.58  167.98 | 8  7 |
|  | sex + season  region + season  TL + sex + region | 169.95  169.46  167.93 | 8  9  8 |
|  | TL + sex + season  TL + region + season  sex + region + season  TL + sex + region + season | 167.03  170.70  171.33  166.85 | 9  10  10  11 |
|  | TL × sex | 167.55 | 7 |
|  | TL × region  TL × season  sex × region | 169.58  179.54  172.37 | 9  11  9 |
|  | sex × season | 175.65 | 11 |
|  | TL × sex × region | 178.99 | 15 |
|  | TL × sex × season | 192.57 | 19 |

The set of best-fit predictors for each parasite is denoted by an asterisk, based on the lowest Akaike’s Information Criterion (AIC) value, or, if lowest AIC values were within two units of each other, the set of predictors with the lowest degrees of freedom and within two units of lowest AIC value.

**Supplementary Table S4** Predictor variables tested for best-fit for predicting the intensity of infection for Caligus furcisetifer, Stibarobdella macrothela and gnathiid pranizae on the giant shovelnose ray, Glaucostegus typus, in Western Australia. A zero-inflated, negative binomial distribution model was used for each species. A ‘+’ denotes an effect of multiple variables; a ‘×’ denotes an effect of multiple variables, including their interaction. Variables retained in models as best-fit predictors of infection presence for each species are as follows: C. furcisetifer, host total length (TL) and sample season; S. macrothela, TL, sample region and their interaction; gnathiid pranizae, sample region.

| Parasite | Predictor | AIC | DF |
| --- | --- | --- | --- |
| *Caligus furcisetifer* | control | 382.31 | 7 |
|  | TL* | 377.29 | 8 |
|  | sex  region | 383.12  382.88 | 8  9 |
|  | season | 387.47 | 10 |
|  | TL + sex  TL + region | 379.24  381.49 | 9  10 |
|  | TL + season  sex + region | 378.60  384.32 | 11  10 |
|  | sex + season  region + season  TL + sex + region | 387.55  380.97  383.47 | 11  12  11 |
|  | TL + sex + season  TL + region + season  sex + region + season  TL + sex + region + season | 380.60  379.89  381.59  381.65 | 12  13  13  14 |
|  | TL × sex | 379.73 | 10 |
|  | TL × region  TL × season  sex × region | 384.65  377.54  385.78 | 12  14  12 |
|  | sex × season | 394.27 | 14 |
|  | TL × sex × region | 391.08 | 18 |
|  | TL × sex × season | 392.41 | 22 |
| *Stibarobdella macrothela* | control | 650.87 | 8 |
|  | TL | 515.24 | 9 |
|  | sex  region | 653.08  560.08 | 9  10 |
|  | season | 641.89 | 11 |
|  | TL + sex  TL + region | 517.19  515.37 | 10  11 |
|  | TL + season  sex + region | 520.33  561.92 | 12  11 |
|  | sex + season  region + season  TL + sex + region | 575.28  559.06  517.33 | 12  13  12 |
|  | TL + sex + season  TL + region + season  sex + region + season  TL + sex + region + season | 522.30  516.75  561.30  519.09 | 13  14  14  15 |
|  | TL × sex | 519.53 | 11 |
|  | TL × region*  TL × season  sex × region | 511.32  522.43  566.21 | 13  15  13 |
|  | sex × season | 580.17 | 15 |
|  | TL × sex × region | 519.37 | 19 |
|  | TL × sex × season | 533.83 | 23 |
| Gnathiid pranizae | control | 214.08 | 5 |
|  | TL* | 165.83 | 6 |
|  | sex  region | 215.75  218.37 | 6  7 |
|  | season | 214.78 | 8 |
|  | TL + sex  TL + region | 170.80  169.54 | 7  8 |
|  | TL + season  sex + region | 164.86  220.09 | 9  8 |
|  | sex + season  region + season  TL + sex + region | 216.68  218.90  171.18 | 9  10  9 |
|  | TL + sex + season  TL + region + season  sex + region + season  TL + sex + region + season | 166.88  169.24  220.94  171.30 | 10  11  11  12 |
|  | TL × sex | 166.80 | 8 |
|  | TL × region  TL × season  sex × region | 173.59  169.30  223.44 | 10  12  10 |
|  | sex × season | 221.35 | 12 |
|  | TL × sex × region | 181.73 | 16 |
|  | TL × sex × season | 184.67 | 20 |

The set of best-fit predictors for each parasite is denoted by an asterisk, based on the lowest Akaike’s Information Criterion (AIC) value, or, if lowest AIC values were within two units of each other, the set of predictors with the lowest degrees of freedom and within two units of lowest AIC value.
